# Post-use assay of vaginal rings (VRs) as a potential measure of clinical trial adherence

**DOI:** 10.1016/j.jpba.2016.03.023

**Published:** 2016-06-05

**Authors:** Patrick Spence, Annalene Nel, Neliëtte van Niekerk, Tiffany Derrick, Susan Wilder, Bríd Devlin

**Affiliations:** a International Partnership for Microbicides, 8401 Colesville Road, Suite 200, Silver Spring 20910, MD, USA; b International Partnership for Microbicides, 63 Main Road, Paarl 7645, South Africa; c Genentech USA, Inc., 1 DNA Way, South San Francisco 94080-4990, CA, USA

**Keywords:** Dapivirine vaginal ring, Post-use assay, Higuchi model, In vivo drug release, In vitro drug release, Adherence model

## Abstract

Adherence measurement for microbicide use within the clinical trial setting remains a challenge for the HIV prevention field. This paper describes an assay method used for determining residual dapivirine levels in post-use vaginal rings from clinical trials conducted with the Dapivirine Vaginal Matrix Ring-004 developed by the International Partnership for Microbicides to prevent male to female HIV transmission. Post-use assay results from three Ring-004 clinical trials showed that of the 25 mg drug load, approximately 4 mg of dapivirine is released from the matrix ring over a 28-day use period. Data obtained by both in vitro and in vivo studies indicate that dapivirine is released according to a diffusion mechanism, as determined by conformance of both data sets to the Higuchi equation. This, coupled with the low variability associated with batch production over two manufacturing sites and 20 batches of material, provides evidence that post-use ring analysis can contribute to the assessment of adherence to ring use. Limitations of this method include the potential of intra-participant and inter-participant variability and uncertainty associated with measuring the low amount of dapivirine actually released relative to the drug load. Therefore, residual drug levels should not serve as the only direct measurement for microbicide adherence in vaginal ring clinical trials but should preferably be used as part of a multi-pronged approach towards understanding and assessing adherence to vaginal ring use.

## Highlights

• Simulated use of dapivirine VRs showed no content changes after storage at RT or −20 °C.
• On average, about 4 mg of dapivirine is released in vivo after 28 days of VR use.
• Both in vivo and in vitro drug release conform to the Higuchi equation.
• Residual ring data should be used in conjunction with other PK measures for modeling adherence.

## 1 Introduction

Topical vaginal microbicides represent a promising prevention technology with the potential to fulfil the need for woman-initiated HIV prevention options. Numerous clinical trials have been conducted over the past 15 years, with vaginal microbicide products containing a range of active pharmaceutical ingredients (APIs), primarily in gel formulation for coitally-associated use. Only one product, a gel formulation of the antiretroviral tenofovir, has demonstrated efficacy in a clinical trial, but results from a subsequent tenofovir trial failed to confirm efficacy [1,2][1,2a]. Increasingly, the inability to demonstrate efficacy of candidate microbicides is attributed, at least in part, to poor user-adherence in large scale clinical trials [2], underlying the importance and need for the development and optimization of robust adherence measures in anti-HIV microbicide trials.

Adherence assessment for vaginal microbicides has proven challenging. As Karim et al. observe, “Adherence levels are dependent on human behavior in the context of the user’s social environment, whereas the available concentration of the drug is affected by its pharmacological properties and host-cell biology.” [3]. Similar to many other health care products, trial participants may over-report microbicide use because of a social desirability bias, due to the possibility that they may not fully understand the questions about their product use, and/or they may not accurately recall use [4].

Self-administered vaginal rings (VRs) are a promising method for delivery of topical antiretroviral microbicides and potentially offer an adherence advantage for women over daily or coitally-dependent dosage forms such as gels. VR dosage forms release the active substance over an extended period of time, often weeks or even months. A vaginal ring developed by the International Partnership for Microbicides (IPM), containing the antiretroviral dapivirine, a non-nucleoside reverse transcriptase inhibitor, is currently in Phase III clinical development. Two pivotal Phase III safety and efficacy trials with this dapivirine vaginal ring (Ring-004) are ongoing with results expected in 2015–2016 [5].

VR design generally falls into two major categories [6]: *Reservoir rings*, which contain an inner “core” of API(s) dispersed or dissolved in a solution, and an outer layer of pure polymer; and *Matrix rings* that are uniform dispersions of API(s) within a polymer matrix. The most common polymers used in both ring types are silicone, ethinyl vinyl acetate, or polyurethane. The IPM vaginal Ring-004 currently in Phase III development is a platinum-catalyzed silicone ring, containing 25 mg of dapivirine in a matrix formulation, designed to achieve release of dapivirine over a 28-day period [7a–c].

Initial clinical trials of the dapivirine VR [7b–d] included analysis of plasma and vaginal fluid drug levels. This body of bioanalytical work indicated release of dapivirine from the ring at safe levels and presence of significant (but variable) levels of dapivirine locally in the vagina, supporting the continued advancement of the 25 mg dapivirine matrix vaginal ring [7c–d]. Because of the high inter- and intra-participant variability of PK results observed in clinical trials with the dapivirine VR, even in tightly-controlled Phase I trials, an alternative, more objective measure is needed to understand the true level of adherent use of the VR and drug release (implying local and potentially systemic absorption) for large-scale clinical trials.

Standard analytical technologies can be used to characterize VRs. Analytical assessments of VRs have, to this point, primarily focused on the critical design aspect of drug release [8]. This paper describes the validation (via a simulated use stability study using simulated vaginal fluid and whole blood) and results obtained from an assay method used for determining post-use residual dapivirine levels in VRs used by women in the clinical trial setting. Furthermore, the utilization of this methodology as a potential tool to measure clinical trial adherence is discussed.

## 2 Materials and methods

Rings were prepared by dispersion of micronized dapivirine in silicone oil. The dispersion was thoroughly mixed in equal amounts in “Part A” and “Part B” Liquid Silicone Rubber (LSR) kits provided by Nusil Technologies, Inc. (Carpertina, CA, USA). The LSR kits contain a proprietary blend of components necessary to reliably and reproducibly form vaginal rings through a platinum-catalyzed process. The mixtures of dispersed dapivirine are combined via static mixer and injected into ring molds. Removal of the inhibitor and polymerization to form the ring matrix occurs upon heating to ca. 185 °C for about one minute.

### 2.1 Preparation of proxy (simulated) vaginal fluid

Proxy (simulated) vaginal fluid was prepared similar to the methodology described by Owen and Katz [9]. The simulated vaginal fluid (pH 4.2) was prepared by accurately weighing the following ingredients into a 500 ml glass beaker: 1.8 g sodium chloride (Fluka, Aston Manor, South Africa) 0.7 g potassium hydroxide (Fluka), 0.11 g calcium hydroxide (Merck, Halfway House, South Africa), 0.2 g urea (Merck), 2.5 g glucose (Merck), 9 mg bovine serum albumin (Amresco, Sloane Park, South Africa) and 0.08 g 100% glycerol (SAARCHEM, distributed by Merck). Approximately 400 ml deionized water and 1 ml of 90% lactic acid (Merck) were added to the beaker. The solution was stirred until all of the salts were dissolved. The pH was then adjusted to 4.2 with a mixture (1:1, v/v) of acetic acid (Merck) and water. The solution was transferred to a 500 ml volumetric flask and the volume was adjusted to exactly 500 ml with deionized water. The proxy-vaginal fluid was stored at 5 °C for a period of 4 months.

### 2.2 Vaginal rings used in simulated use studies and clinical trials

The VRs used for simulated use studies and for clinical trial supply were manufactured for IPM by QPharma (Malmö, Sweden).

### 2.3 Simulated use and assay of vaginal rings

#### 2.3.1 Preparation of simulated post-use vaginal rings

Unused VRs (25 mg dapivirine per ring) were subjected to post-use storage and transport conditions similar to VRs from ongoing Phase III clinical trials prior to analysis (e.g., storage in a sealed plastic bag at either −20 °C or room temperature). These VRs were tested for appearance and assay only. Acceptable stability at room temperature and −20 °C has been demonstrated for 6 months and 24 months, respectively.

#### 2.3.2 Simulated use conditions

Simulated use conditions were performed to support the lack of impact of biological fluids on the post-use extraction/analysis, and to probe the effect of not rinsing the rings prior to extended storage (i.e., for shipment to the bioanalytical laboratory for analysis). The following conditions were investigated at both room temperature, up to 6 months, and −20 °C, up to 24 months.

##### 2.3.2.1 Whole blood, rinsed

VRs were submerged in fresh (never frozen) pooled whole blood (anti-coagulant, K3-EDTA), rinsed under tap water, blotted dry, and sealed into appropriately labelled plastic bags. Rings were stored at room temperature and −20 °C.

##### 2.3.2.2 Whole blood, not rinsed

VRs were subjected to the same procedure as described above, but the rings were not rinsed. Rings were stored at room temperature and at −20 °C.

##### 2.3.2.3 Proxy (simulated) vaginal fluid, rinsed

VRs were submerged in proxy-vaginal fluid for, rinsed under tap water, blotted dry and sealed into appropriately labelled plastic bags. Rings were stored at room temperature and −20 °C.

##### 2.3.2.4 Proxy (simulated) vaginal fluid, not rinsed

VRs were subjected to the same procedure as described above, but the rings were not rinsed. Rings were stored at room temperature and −20 °C.

##### 2.3.2.5 Control rings

VRs were not subjected to any treatment. They were sealed in appropriately labelled plastic bags. Rings were stored at room temperature and −20 °C.

#### 2.3.3 Sample handling for post-use ring analysis

Prior to receipt in the laboratory, used rings were collected at the clinical Phase I unit, stored for up to one month at room temperature (if storage for longer than 1 month was required, rings were transferred to a −20 °C freezer), then sent to the laboratory for analysis. Samples were examined for visible microbial contamination prior to delivery to the analytical laboratory. Rings that showed visible signs of microbial contamination were discarded without testing.

#### 2.3.4 Assay methodology

Analysis was conducted using acetone extraction, and high-pressure liquid chromatography (HPLC) similar to the procedure described by Lyndgaard et al. [10]. Minor modifications, such as changing from multi-point to single point calibration were performed, following appropriate crossover or supplemental validation studies. The method was validated for linearity and accuracy over the range of approximately 0.2 mg/ring–25 mg/ring. Selectivity, precision, robustness, and system suitability were also evaluated and results passed expected acceptance criteria.

## 3 Results and discussion

### 3.1 Simulated use testing

The methodology of choice for determination of dapivirine content in used VRs is similar to the assay method initially used to release the clinical drug product following manufacturing. In order to test the applicability of the methodology, the drug product VR was subjected to simulated use conditions, i.e., exposure to simulated vaginal fluid or whole blood, followed by extraction, analysis and comparison to results from the control VR using identical methodology. In order to validate the potential holding times and to assess long-term impact of exposure to fluids and rinsing processes, simulated used rings were stored for 6 months at room temperature (20–25 °C) and 24 months at −20 °C. Results from simulated use studies are presented in Table 1. No visible signs of microbial growth were noted in any of the samples stored at either condition.

With the exception of the 3-month time point (where a sample handling error occurred), data are consistent with the hypothesis that rings can be stored either at room temperature or in the freezer for extended periods prior to analysis with no impact on the assay value.

Similar to matrix transdermal delivery systems, matrix VRs are not designed to release the entire drug loaded into the matrix. Drug delivery happens through a diffusion-controlled system (Eq. (1)), and in vitro drug release models the Higuchi equation.

Eq. (1):

$$Q={\left[D_{m}(2A-C_{p})C_{p}t\right]}^{1/2}$$

where *Q* is the cumulative release per unit area (mg cm^−2^), *D_m_* is the apparent diffusion coefficient (cm^2^ day^−1^) of the substance through the polymer matrix, *A* is the loading per unit volume (mg cm^−3^), *C_p_* is the solubility of the substance in the polymer matrix (mg cm^−3^), and *t* is time (days).

**Fig. 1:**
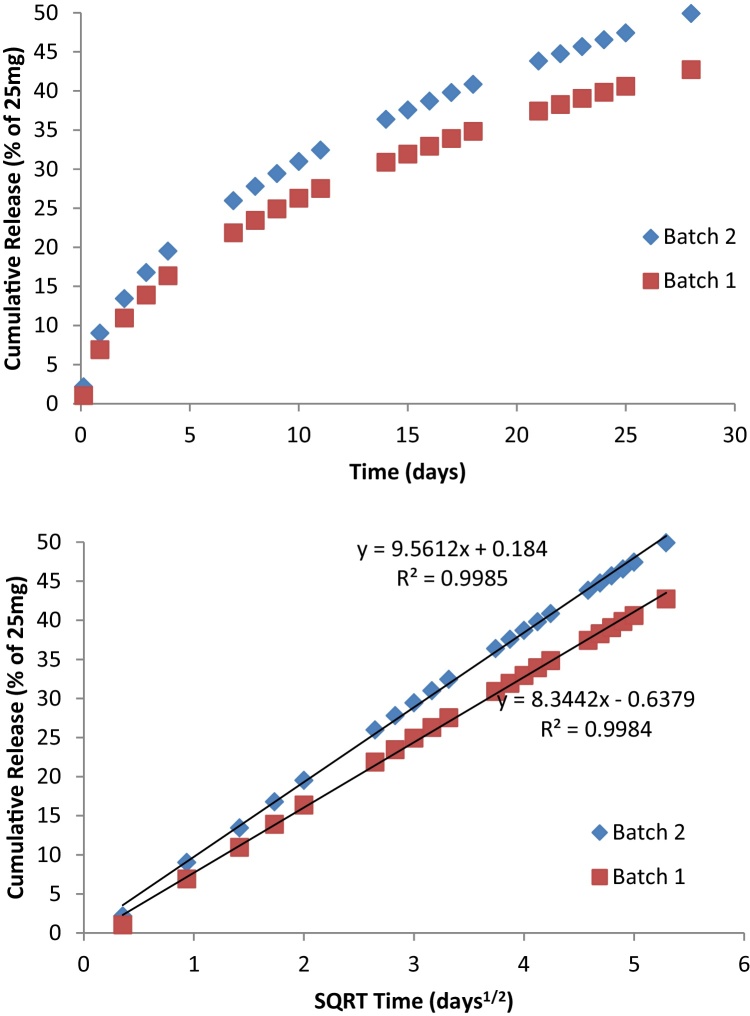
Representative in vitro drug release for the dapivirine ring. QC Method for dissolution: 50:50 2-propanol:water, 37 °C, orbital shaker at 60 rpm with 100 ml media, replaced daily (200 ml replacement on Fridays as sampling did not occur on the weekends). Cumulative release percent is based on 25 mg loading of ring.

**Fig. 2:**
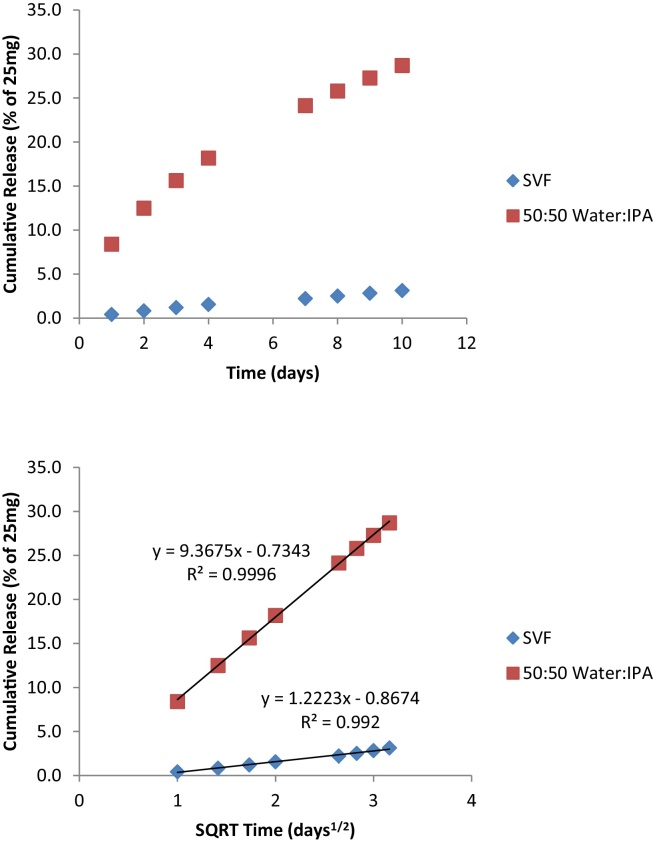
Release of dapivirine in simulated vaginal fluid (SVF) media as compared to the media described in the QC method. Dissolution conditions: simulated vaginal fluid or 50:50 2-propanol:water, 37 °C, orbital shaker at 60 rpm with 100 ml media, replaced daily (200 ml replacement on Fridays as sampling did not occur on the weekends).

“Batch 1” and “Batch 2” (Fig. 1) represent the extremes of the rates of release observed in the 25 mg dapivirine matrix vaginal rings produced to date, which spans over 20 lots at two different manufacturing sites. F_2_ similarity is demonstrated at all time points tested, as evidenced by the maximum absolute percent difference of 7.2%, noted at the 28-day time point. In simulated vaginal fluid, the release of dapivirine also appears to model the Higuchi equation; although the amount released is significantly lower (Fig. 2), clearly a result of the lower dapivirine solubility in this media. The amount of dapivirine was not measurable in the media after 10 days.

From an analytical perspective, the amount of residual dapivirine in used rings provides an indication of how much dapivirine was released over the use period. However, the residual amount of dapivirine will vary, depending on the conditions and duration of use, where “use” can be defined as either participant use per protocol (e.g., vaginal insertion, worn for 28 consecutive days), or any exposure condition for any specified period of time (e.g., in vitro release testing where the ring is submersed in a solution for 28 days). The amount of dapivirine remaining in the ring can be measured after either of these types of exposure conditions by subtracting the amount remaining in the ring from the amount of drug that was initially loaded into each ring, measured prior to batch release.

Such an assessment must consider factors that can influence (1) the *amount* of dapivirine *released* into the surrounding environment, which would typically be the same factors that would influence the amount of drug remaining in the ring; as well as (2) the *measurability* of the amount of drug in the ring before and after use.

Factors that can potentially influence the amount of dapivirine released in the surrounding environment (or the amount of dapivirine remaining in the ring) include changes in the continuous use of the product (removing the ring from the surrounding environment), direct or indirect contact with the surrounding environment, and combinations of these. When tested in vitro, the contact with the surrounding environment (e.g., dissolution or extraction solvent) is continuous, and set-up to provide sink conditions, so that any release of dapivirine into the surrounding environment is not impeded or impacted by the surrounding environment (i.e., the solubility of dapivirine in release media).

This is not the same for actual in-use conditions. In actual human use conditions, the direct or indirect contact can be affected by insertion of the ring, so that the ring may potentially end up with a less than maximum ring surface area contact with the vaginal walls. The impact of this difference on the amount of drug released has not been assessed; however, variation in insertion is minimized by providing all users with standardized instructions for ring insertion and by research staff checking the correct positioning of the ring. Nevertheless, even if the same ring position in the vagina among all users for every executed insertion is assumed, there are still physical differences that may impact the surface area contact. It is not possible to identify or quantify all possible combinations of differences between users, any combinations of which may impact release of product during use. Furthermore, the ring may be out of the vagina, due to expulsion or intentional removal. This would expose the ring to a different environment during these periods, thus potentially affecting the total amount of dapivirine left in the ring. Other factors that could influence the dapivirine released from the ring include rinsing or washing the ring [11], and storing it for an unspecified period of time in conditions of unknown sanitation. Another potential factor for differences in the amount of dapivirine released (inter-participant variability) during actual use is the difference in the vaginal environment itself. Basal body temperatures, pH, bacterial microenvironments, etc., could affect the body’s ability to remove the dapivirine from the matrix (via either diffusion or extraction mechanisms) and may be influenced by a number of factors such as fever, illness, infections (sexually transmitted and non-specific infections), etc. Furthermore, women’s use of a range of vaginal products to improve sexual intercourse, or for cleaning before or after sex or menses, could affect the vaginal environment [12].

Factors that can impact the measurability of the amount of dapivirine in the ring before and after use include variability in the initial drug load, variation due to the analytical method used to determine the residual amounts and variation due to in-use conditions. Although the target drug load for all rings is 25 mg dapivirine per ring, current specifications allow for drug load ranging from 90.0% to 110.0% of the target label claim (25 mg). Therefore, an acceptable ring may contain anywhere from 22.5 mg to 27.5 mg dapivirine. Although this degree of variability is allowed for release of product, the actual variability, as one would expect for a late-phase pharmaceutical drug product is significantly less. Across 20 batches manufactured using the current process, the average assay value is 98.0% label claim (or 24.5 mg/ring), with a 1.87% RSD. Variation may be due to the analytical method used to determine the residual amounts. The analytical method used to extract any remaining dapivirine from rings was validated under “clean”/non-clinical use conditions; therefore, one must assume that in the post-use scenario there is as much as the standard accepted 2% potential variation due to the combined method and equipment variables.

Variations due to in-use conditions might impact this 2%, since after rings are used and removed they are rinsed and patted dry, then placed into a pouch where they are stored until analysis. While stored at the research centers, these returned rings are stored at ambient conditions. Occasionally, biological growth has been observed on some of the rings between the time of storage and the time of analysis, so it would theoretically be possible that exposure to biological fluids and potential biological growth on the ring may impact the ability to extract all remaining dapivirine from the ring during analysis. To address this potential, the study described in Section 2 was completed and the results indicated that there was no change in extracted amounts of drug versus expected amounts of drug; however, there was also no biological growth observed, so the impact of visible microbial growth on post-use VR assay could not be determined.

### 3.2 Potential use of residual drug analysis as a measure of adherence in clinical trials

Results on dapivirine residual levels in post-use rings from three clinical trials of the dapivirine vaginal ring (Ring-004), conducted by IPM, is presented in Table 2. On average, about 4 mg of dapivirine was released in vivo over the course of 28 days. In the Phase I/II trial, IPM 015, this corresponded to trough plasma levels (as determined at the time of ring removal) typically <300 pg/ml, explained mechanistically by the transitioning of the drug from the vaginal mucosa through barrier layers (i.e., epithelial) prior to entering the bloodstream. Similar results were obtained for the Phase I clinical trials, IPM 028 and IPM 034 that were both conducted at a Phase I unit in Belgium. Due to the better control of these trials (by virtue of more frequent clinical visits), it is expected that adherence would be higher than might be seen in larger trials.

**Fig. 3:**
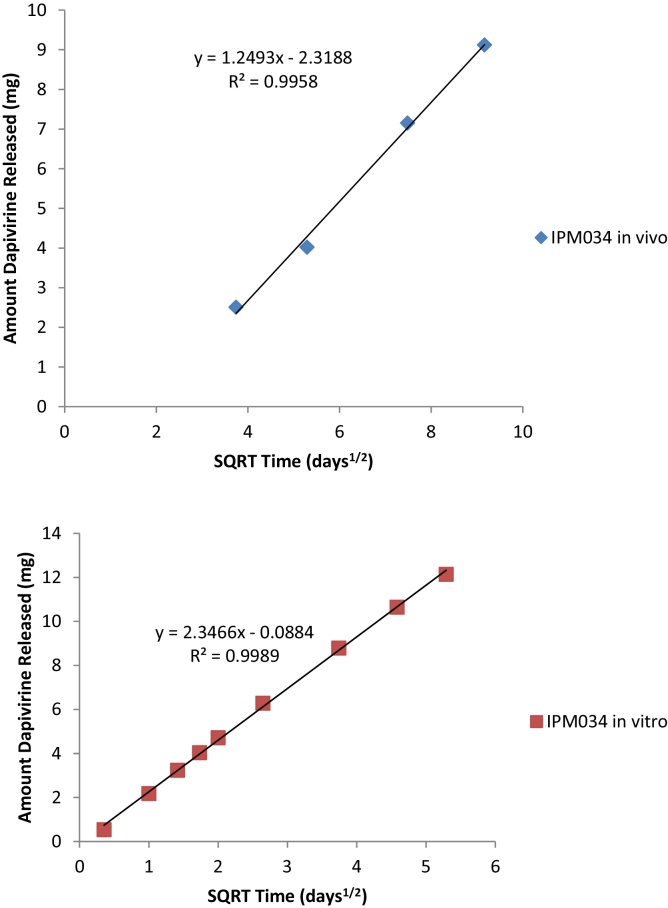
Comparison of in vivo and *in vitro* data for IPM 034. In vitro release conditions: 50:50 2-propanol:water, 37 °C, orbital shaker at 60 rpm with 100 ml media, replaced daily (200 ml replacement on Fridays as sampling did not occur on the weekends).

Results from the more recent Phase I trial, IPM 034, indicate that a correlation exists between the amount of residual dapivirine and the duration of ring use (Fig. 3); therefore, one would expect that it would be theoretically possible to model and predict adherence based on observed ring residual amounts, given a significant sample size.

The original design for the IPM 034 trial included a 7-day duration of ring use; however, the data from this group are excluded from consideration in this trend analysis due to analytical errors. Although the number of rings analyzed for the three trials described in Table 2 ranged from 32 to 331, the residual dapivirine level data were observed to be within 10% of each other at the 28-day time point. This provides the basis for a prediction that the dapivirine ring delivers, on average, about 4 mg of dapivirine over a 28-day use period. Moreover, as different batches of rings were used for the three clinical trials, and manufactured at two different sites, these results support that the technology used to manufacture the rings provides reproducible, potentially predictable drug product, confirming the trends in similarity observed between lots in the in vitro dissolution comparison presented in Fig. 1.

When plotted against the square root of time, both the in vivo and in vitro data for the lot used in the clinical trial IPM 034 conform to the Higuchi equation, with linear coefficients of determination calculated as 0.9958 and 0.9989, respectively (Fig. 3). This further implies predictability by demonstrating that the in vivo release is controlled primarily via a diffusion mechanism, similar to what was observed in vitro. Despite the fact that all of the processes that contribute to in vivo release mechanisms cannot be known, this key comparison shows promise that residual drug level can be used as one predictive tool for clinical trial adherence. Limitations of the ability to predict directly from used ring data alone are realized in that:

1. Inter-participant variability is seen in post-use ring values and outliers are observed at both the high (low drug release) and low (high drug release) end of the residual drug level spectrum measured. In larger clinical studies (i.e., IPM 015 in this paper) there is a larger incidence of outliers observed for residual drug values that are greater than two standard deviations above the average, presumably due to lower adherence to product use in these participants.
2. For larger trials, analytical variability may play an important role, in regards to the number of rings analyzed and the labor intensive nature of the sample preparation (e.g., in this case, 24-h extraction, followed by HPLC analysis). Based on the sheer number of potential factors that can influence the amount of dapivirine released in vivo (vide supra), it is not possible to specify an exact amount of dapivirine to be released for each participant. Since there is no “target” value for post-use ring assays, description of the overall variability can only be realistically achieved by conducting a statistical analysis on a large data set of used rings and flagging/investigating future values that fall outside predefined “outlier” criteria.
3. Given the small amount of dapivirine that is released (about 4 mg over a 28-day ring use period), the error inherent in the analytical method itself, typically accepted as around 2% of the label claim of 25 mg (taking into account method and equipment variability), corresponds to as much as 0.5 mg/ring.

It appears that pooled, blinded post-use residual dapivirine level data can provide an initial, general trend of some or no product use in vaginal ring clinical trials. Despite the many variables that make it not possible to rely on these values alone, a more comprehensive indication of adherence from post-use residual dapivirine levels can be triangulated with data from plasma or other pharmacokinetic parameters (i.e., vaginal fluid, or other parameters) to provide an objective measure of adherence during the conduct of Phase III trials.

## 4 Conclusion

Validation (via simulated use) and stability studies have been performed on methodology used to determine dapivirine levels in post-use vaginal rings, with the potential purpose of assessing clinical trial adherence. Samples rinsed in either whole blood or simulated (proxy) vaginal fluid were stored with or without rinsing for up to 6 months at room temperature (20–25 °C) and 24 months at −20 °C, and no significant differences in assay value were observed.

Data obtained by both in vitro and in vivo studies indicate that dapivirine is released according to a diffusion mechanism, by virtue of conformance of both data sets to the Higuchi equation. This, coupled with the low variability associated with batch production over two sites and 20 batches of VRs [13], provides insight that post-use ring analysis could be used as one tool in predicting adherent use of VRs in clinical trials. Potential inter-participant variability and uncertainty associated with measuring the low amount of dapivirine actually released (about 4 mg over a 28-day ring use period) relative to the starting amount (about 25 mg) are limitations on the sole use of residual ring values as direct measurements for adherence in vaginal ring clinical trials. A comprehensive model to describe adherence, based on ring residual amounts and data from plasma and vaginal fluids, will be proposed in future work, once the pivotal Phase III trials for the dapivirine vaginal ring are completed.

## Sources of financial support

The clinical trials described in this manuscript were funded by the International Partnership for Microbicides (IPM) (a not-for-profit public-private partnership). This work was made possible through generous support from the United Kingdom Department for International Development (DFID) and the Bill and Melinda Gates Foundation.

## Disclaimers

The contents of this article are the responsibility of IPM and do not necessarily reflect the views of its donors.

## Conflicts of interest

IPM provided the candidate microbicide and vaginal placebo rings used in the clinical trials. The authors are all current or former employees of IPM. With the exception of the above disclosure, the authors declare no other conflicts of interest.

## Figures and Tables

**Table 1:** Results of simulated use studies: 24-month data.

Description	Condition	Time Point (months)	Assay (% LC, mean)^a^	RSD^b^ (%, n = 3)
Control	N/A	0	100.8	0.6
	Room Temperature	6	98.6	N/A^c^
	−20 °C	6	99.2	0.4
	Room Temperature	12	99.1	0.2
	−20 °C	12	99.5	0.6
	Room Temperature	24	100.8	0.7
	−20 °C	24	99.5	0.8

SVF, rinsed	N/A	0	100.7	0.1
	Room Temperature	3	103.1^d^	0.2
	−20 °C	3	101.3	0.4
	Room Temperature	6	100.0	0.8
	−20 °C	6	99.2	0.4
	−20 °C	12	99.1	0.8
	−20 °C	24	100.5	0.9

SVF, not rinsed	N/A	0	100.6	0.4
	Room Temperature	3	102.0^d^	0.5
	−20 °C	3	100.7	0.8
	Room Temperature	6	99.3	0.5
	−20 °C	6	98.3	0.8
	−20 °C	12	98.7	1.0
	−20 °C	24	99.3	0.8

Whole blood, rinsed	N/A	0	100.5	0.1
	Room Temperature	3	105.0^d^	0.8
	−20 °C	3	104.5^d^	1.9
	Room Temperature	6	99.2	0.7
	−20 °C	6	97.6	1.2
	−20 °C	12	97.5	0.9
	−20 °C	24	99.6	1.1

Whole blood, not rinsed	N/A	0	100.9	0.8
	Room Temperature	3	102.2^d^	0.5
	−20 °C	3	103.9^d^	1.4
	Room Temperature	6	99.5	1.2
	−20 °C	6	99.6	0.4
	−20 °C	12	98.5	0.8
	−20 °C	24	101.6	1.4

a Label Claim (LC) based on 25 mg theoretical dapivirine amount.

b Relative Standard Deviation.

c Not Applicable—mean assay value based on n = 2 due to analytical error.

d Atypical values were obtained at the 3-month time point of the study. Results of the investigation performed indicated that the high values obtained were likely due to improper sample handling, and are disregarded for trending purposes.

**Table 2:** Residual dapivirine levels in post-use vaginal rings from clinical trials.

Clinical Trial	Duration of ring use (d)	Average drug released (mg)^a^	SD	Sample size (N)^b^
IPM 015	28 ± 4	4.4	1.6	331
IPM 028	28	4.4	1.5	67
IPM 034	14	2.5	0.3	8
	28	4.0	0.6	8
	56	7.1	0.8	8
	84	9.1	1.2	8

a Lot average value at release (mg)—value obtained from post-use assay (mg). Average dapivirine released does not include removal of statistical outliers.

b Number of rings analyzed.
